# A Study on Microwave Absorption Properties of Carbon Black and Ni_0.6_Zn_0.4_Fe_2_O_4_ Nanocomposites by Tuning the Matching-Absorbing Layer Structures

**DOI:** 10.1038/s41598-020-60107-1

**Published:** 2020-02-21

**Authors:** Idza Riati Ibrahim, Khamirul Amin Matori, Ismayadi Ismail, Zaiki Awang, Siti Nor Ain Rusly, Rodziah Nazlan, Fadzidah Mohd Idris, Muhammad Misbah Muhammad Zulkimi, Nor Hapishah Abdullah, Muhammad Syazwan Mustaffa, Farah Nabilah Shafiee, Mehmet Ertugrul

**Affiliations:** 1 Materials Synthesis and Characterization Laboratory, Institute of Advanced Technology, Universiti Putra Malaysia, 43400 UPM Serdang, Selangor Malaysia; 2 Department of Physics, Faculty of Science, Universiti Putra Malaysia, 43400 UPM Serdang, Selangor Malaysia; 3 Microwave Research Institute, Universiti Teknologi MARA, 40450 Shah Alam, Malaysia; 4 Department of Materials Technology, Faculty of Industrial Science and Technology, Universiti Malaysia Pahang, Kampus Gambang, Lebuhraya Tun Razak, Kuantan, Pahang Malaysia; 5 GENIUS Insan College, Universiti Sains Islam Malaysia, PERMATA Insan Complex, Bandar Baru Nilai, 71800 Nilai, Negeri Sembilan Malaysia; 6 Functional Devices Laboratory, Institute of Advanced Technology, Universiti Putra Malaysia, 43400 UPM Serdang, Selangor Malaysia; 7 Ataturk University, Engineering Faculty, Department of Electrical and Electronics Engineering, 25240 Erzurum, Turkey

**Keywords:** Energy science and technology, Materials science, Nanoscience and technology, Physics

## Abstract

Microwave absorption properties were systematically studied for double-layer carbon black/epoxy resin (CB) and Ni_0.6_Zn_0.4_Fe_2_O_4_/epoxy resin (F) nanocomposites in the frequency range of 8 to 18 GHz. The Ni_0.6_Zn_0.4_Fe_2_O_4_ nanoparticles were synthesized via high energy ball milling with subsequent sintering while carbon black was commercially purchased. The materials were later incorporated into epoxy resin to fabricate double-layer composite structures with total thicknesses of 2 and 3 mm. The CB1/F1, in which carbon black as matching and ferrite as absorbing layer with each thickness of 1 mm, showed the highest microwave absorption of more than 99.9%, with minimum reflection loss of −33.8 dB but with an absorption bandwidth of only 2.7 GHz. Double layer absorbers with F1/CB1(ferrite as matching and carbon black as absorbing layer with each thickness of 1 mm) structure showed the best microwave absorption performance in which more than 99% microwave energy were absorbed, with promising minimum reflection loss of −24.0 dB, along with a wider bandwidth of 4.8 GHz and yet with a reduced thickness of only 2 mm.

## Introduction

In order to address issues induced by high proliferation of electromagnetic interferences in both civil and military applications, efficient microwave absorbers are becoming highly desirable and necessary. For that reason, such material is required to effectively reduce the reflection of electromagnetic (EM) signals over a broad absorption bandwidth. In order to improve the performance of microwave absorption properties, microwave absorbers are designed to meet the specific requirements of simultaneously having strong absorption, wide frequency band, lightweight and small thickness. Improvements can certainly be made to the designs by physical assembling of different types of absorbents^1–5^, chemical decorated absorbents^6,7^ as well as by designing multi-layer structures^8–11^.

Microwave absorbers are produced using different kinds of materials including one dimensional (1D) materials such as carbon nanotubes^12–15^, two dimensional (2D) materials such as graphene^16,17^ and bulk three dimensional (3D) materials such as ferrites^9,18–21^. The difference in the dimensional structure of the materials would largely affect the microwave absorption performances since different kinds of structures contribute to different surface area which then determines interfacial polarization, and the scattering and reflection of EM waves between the surfaces.

Two main pre-requisites in the design of a microwave absorber are dielectric and magnetic losses. Ferrite is a very important class of magnetic material, in particular a metal oxides containing magnetic ions arranged in such a way that it produces spontaneous magnetization, while having good dielectric properties^20^. Ferrites absorb electromagnetic wave energy by hysteresis loss and ferromagnetic resonance. NiZn ferrite, a type of ferrite, has been commercially used as electromagnetic devices operated at high frequencies (>10 MHz) – its popularity arising from the fact that metal dopants can be used to modify the electromagnetic properties by increasing the resistivity and permeability. Instead, this spinel ferrite typically displays relatively low permittivity which is much lower than those of magnetic metal materials and possesses high electrical resistivity^22^. Therefore, these parameters are useful features to make good matching layer which facilitate an incident EM wave entering the absorber. Particularly, the magnetic performance of NiZn ferrite (Ni_x_Zn_1−x_Fe_2_O_4_) varies with the amount of *x* which is important in determining the absorption properties. Thus, NiZn ferrite with the composition of Ni_0.6_Zn_0.4_Fe_2_O_4_ is selected as potential microwave absorbing material since it exhibits high saturation magnetization^23–25^. Moreover, ferrite-polymer composites are useful as absorbers due to their lightweight, low cost, and good design flexibility^26^.

Owing to the conductive loss characteristics, carbon-based materials are largely utilized as potential microwave absorbers^4,7,14,17,27–29^. Even though carbon black employed in this study is a good absorber of electrical energy, the microwave absorbing property of carbon black is not so good due to its non-magnetic nature. Therefore, if carbon black is compounded with ferrite materials, the composite materials will have contributions from dielectric loss and magnetic loss at the same time, thus improving the microwave absorbing property.

The motivation of designing double-layer absorbers is due to the limited number of parameters and narrow absorption bandwidth exhibited by single-layer designs. Furthermore, single-layer absorbers are normally narrowband. Therefore, the development of double-layer absorbers is devised in order to fully utilize the properties of different layers used in absorbing at a wider bandwidth, therefore achieving optimal absorption properties in the structure. Two important conditions should be satisfied in order to have a good absorbing material^30^. The first is the “impedance matching”, in which the intrinsic impedance of the material is made equal to the impedance of the free space. Second, the incident electromagnetic wave must enter and get rapidly attenuated through the material. Double-layer structures can be formed by integrating the matching and absorbing layers, therefore achieving broadband operation conditions is easier. In this study, a similar material has been utilized as both matching and absorbing layers. Therefore, the choice of material for the particular layer is important owing to the impedance matching requirement, which would be explored in this study. When the material is placed in front of the incident EM wave, the material acted as a matching layer and permits the incident EM wave into the material. When the material is placed in another layer behind the matching layer, it acts as an absorbing layer. In this arrangement, when the EM wave is allowed to enter by a matching layer, absorption of EM wave takes place in the following absorber layer which attenuates the incident EM radiation. Therefore, low real part of complex permittivity is desired to facilitate an incident EM wave entering into the absorbing layer. By creating a matching layer with little absorption capacity, the absorber can achieve good impedance matching, and by then adding an absorption layer, we can achieve a good absorption capacity. The functions of the layer could be switched from matching to absorbing layer and vice versa based on the basis of the dielectric and magnetic characteristics exhibited in the samples such that the relative permittivity and relative permeability must be equal to transmit the EM wave without any reflection^31^. Then to become an absorbing layer, EM wave is dissipated through magnetic and dielectric losses of the layer.

Therefore, the objective of this work was to design double-layer absorbers which consisted of NiZn ferrite and carbon black layers and to study the microwave absorption properties by combining the magnetic characteristics of ferrite and dielectric characteristics of carbon black to achieve a broadband performance. Further in our study, the influence of matching and absorbing layer positions was evaluated to see their effect on microwave absorption. Therefore, absorbers with broadband optimal impedance match and highest attenuation can be determined.

## Materials and Methods

The carbon black samples utilized in this study were obtained commercially (TIMCAL C-NERGY™ SUPER C65 Carbon Black), while the NiZn ferrite with the composition of Ni_0.6_Zn_0.4_Fe_2_O_4_ was synthesized in our laboratory using solid state reaction. The starting raw powder materials to produce Ni_0.6_Zn_0.4_Fe_2_O_4_ were mixed according to Eq. (1). The raw materials were high energy ball milled using a *SPEX8000D* mechanical alloying machine for 6 hours and sintered at 900 °C.

1 $$0.6{\rm{NiO}}+0.4{\rm{ZnO}}+{{\rm{Fe}}}_{2}{{\rm{O}}}_{3}\to {{\rm{Ni}}}_{0.6}{{\rm{Zn}}}_{0.4}{{\rm{Fe}}}_{2}{{\rm{O}}}_{4}$$

**Figure 1:**
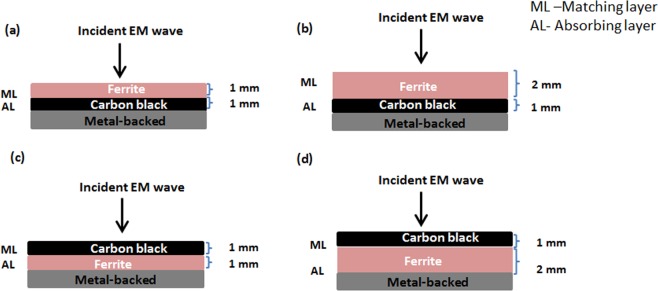
Double-layer structure of ferrite/carbon black composites with (**a**) 1 mm ferrite/1 mm carbon black (F1/CB1), (**b**) 2 mm ferrite/1 mm carbon black (F2/CB1) and (**c**) 1 mm carbon black/1 mm ferrite (CB1/F1) and (**d**) 1 mm carbon black/ 2 mm ferrite (CB1/F2) thickness. In the diagrams, ML and AL are abbreviations for matching and absorbing layers, respectively.

Single layer Ni_0.6_Zn_0.4_Fe_2_O_4_ composites with thicknesses of 1 and 2 mm were made by incorporating 60 wt.% Ni_0.6_Zn_0.4_Fe_2_O_4_ into 40 wt.% epoxy resin and hardener (known as F). The mixture was solution casted into moulds with inner dimension of 23 × 10 mm and 15 × 7 mm for X- and K_u_-band frequency measurements respectively and left to dry for one day at room temperature. To produce a double-layer structure, 5 wt.% of carbon black was incorporated into 95 wt.% of epoxy resin and hardener (known as CB) and poured onto the previously dried Ni_0.6_Zn_0.4_Fe_2_O_4_ layer_,_ thus producing double-layer structure with total thickness of 3 mm. For better picture and explanation of the composite structures and their nomenclature, the layering structures are shown in Table 1 and Fig. 1.

**Table 1:** Details on the double-layer structure of ferrite/carbon black composites.

Sample name	Matching layer	Thickness (mm)	Absorbing layer	Thickness (mm)
F1/CB1	Ferrite	1	Carbon black	1
F2/CB1	Ferrite	2	Carbon black	1
CB1/F1	Carbon black	1	Ferrite	1
CB1/F2	Carbon black	1	Ferrite	2

The phase analysis was carried out by a *Philips X’pert Diffractometer* from 20° to 80° 2θ position, using CuKα radiation source with λ = 1.5418 Å. The microstructural characterization was obtained using a *NovaNano 230* FESEM machine. The particle size distributions were determined by the line intercept method of more than 200 grains per sample and the analysis was carried out by *Image J* software. Electromagnetic characterizations and microwave absorption measurements were carried out using an *Agilent PNA* N5227A network analyzer in a rectangular waveguide using the transmission line method in the frequency range of 8–12 GHz (X-band) and 12–18 GHz (K_u_-band). The complex permittivity and complex permeability values were computed from scattering parameters (S-parameters) using *Agilent Technologies 85071E* Materials Measurement Software. The influence of matching and absorbing layer position on the electromagnetic and microwave absorbing properties were studied by tuning the positions of the ferrite and carbon black layers consecutively.

## Results and Discussion

### Morphological analysis

**Figure 2:**
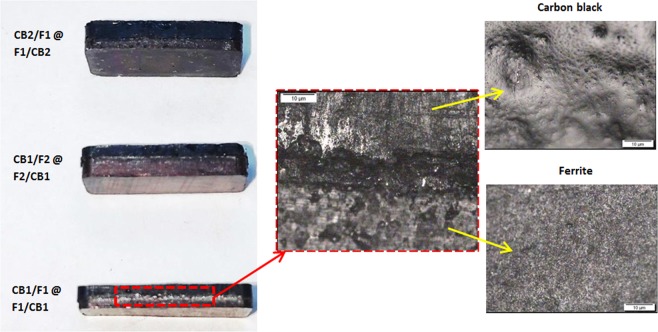
Optical images of the double-layer samples.

Images of a double-layer structure with the inset optical micrograph image of 5× magnification (Fig. 2) illustrate the boundary between the carbon black and ferrite layers. The thickness of the boundary was about 18 µm in which the upper layer is the carbon black layer and the bottom layer is the ferrite layer. The carbon black layer shows a rougher and more porous surface as compared to that of the ferrite layer.

**Figure 3:**
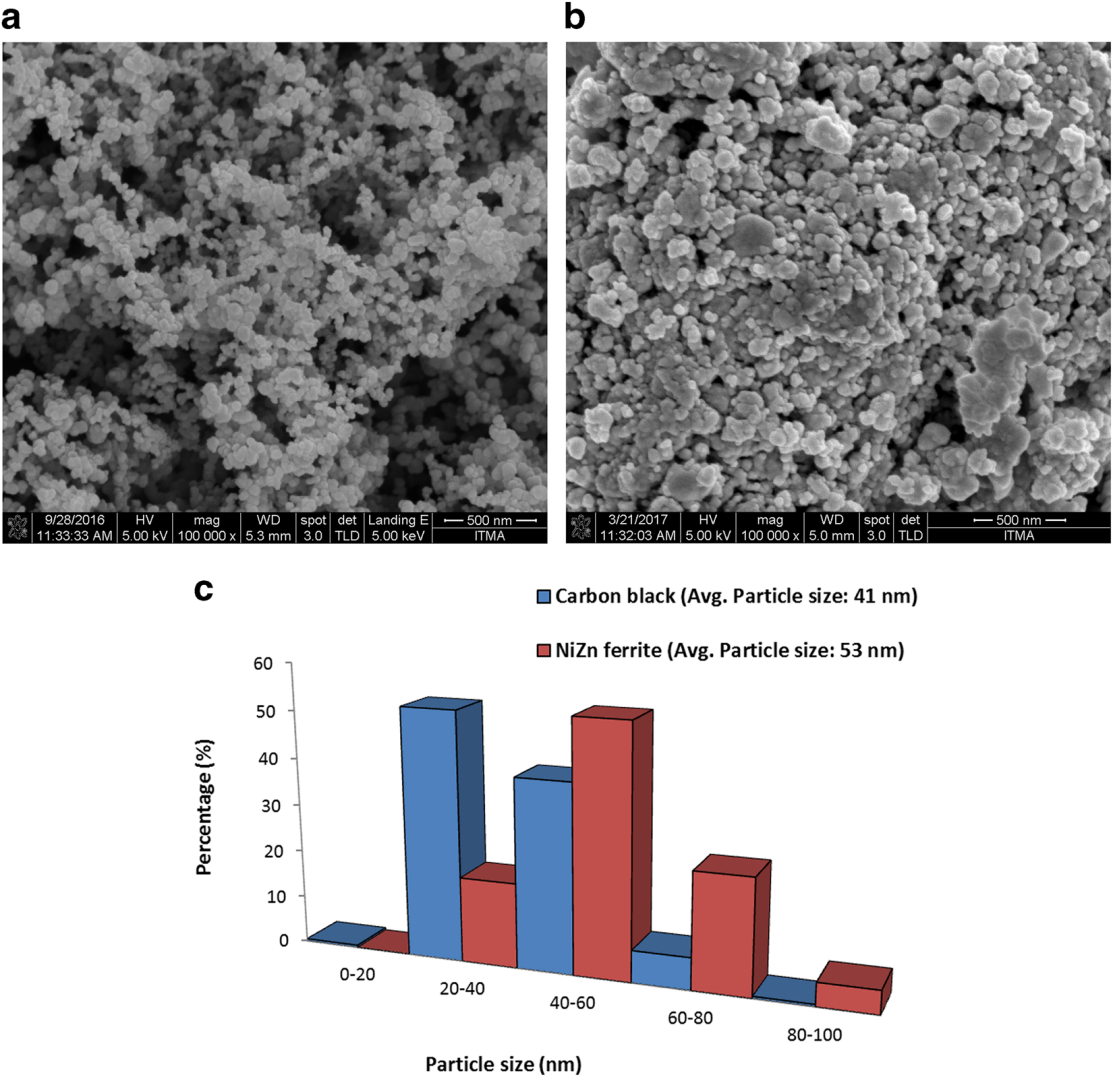
FESEM micrographs of (**a**) carbon black, (**b**) Ni_0.6_Zn_0.4_Fe_2_O_4_ and their (**c**) particle size distributions.

FESEM micrographs and particle size distributions in Fig. 3 show the samples consist mainly of nanoparticles with average particle sizes of 41 and 53 nm for carbon black and Ni_0.6_Zn_0.4_Fe_2_O_4_ respectively. The particle size distribution for carbon black ranged from 10 to 90 nm with spherical shape form. Generally, higher conductivities of the polymer composites can be obtained by using carbon black of smaller particle size (which translates to larger surface area), lower particle density (higher particle porosity), higher structure (better aggregation) and low volatility (fewer chemisorbed oxygen groups)^27^. While for Ni_0.6_Zn_0.4_Fe_2_O_4_, it was observed from the micrographs that the microstructure was highly aggregated with a non-uniform microstructure which resulted from the high energy ball milling process. The particle size distribution ranges from 20 to 100 nm. A previous study^32^ has stated that the critical size for the transition from multi domain to single-domain particle/grain of Ni_0.6_Zn_0.4_Fe_2_O_4_ is 0.40 µm (400 nm). Based on the particle size distribution found in our samples, it shows that the value was below the critical size and we concluded that the samples comprised of single-domain particles with no domain wall. With the reduction in particle size, the surface effects become more obvious, consequently the magnetic and microwave absorption properties differ significantly from those of bulk materials^33^. Due to broken super-exchange bonds, surface spins of ferrite nanoparticles are in a disordered state^34^, thus leading to high magnetic loss. Within a single-domain, the particles are subjected to size-shape anisotropy, strain induced anisotropy and magneto-crystalline anisotropy^35,36^. In addition, the anisotropic energy of small size particles, especially at nanometer scale, may be increased remarkably due to the surface anisotropic field arising from the small size effect. This is due to the influence of anisotropy of defective surface layers, which increased with decreasing particle size. The effective anisotropy constant for uniaxial magnetic nanoparticles can be written as^37^:

2 $${k}_{eff}={k}_{1}+{k}_{f}+{k}_{s}(\frac{{V}_{s}}{V})$$

where ${k}_{1}+{k}_{f}$ is the sum of the contributions from the magnetocrystalline anisotropy and shape anisotropy of the particles, ${k}_{s}$ is the contribution of surface anisotropy, ${V}_{s}$ is the volume of the defective surface layer and *V* is the volume of the particle. The large surface area with variation of particle distribution is expected to show broad absorption, which will lead to several different resonant frequencies^38^. Hence, nanometer particle-sized absorbers are expected to offer wider absorption bandwidths with less reflection losses.

**Figure 4:**
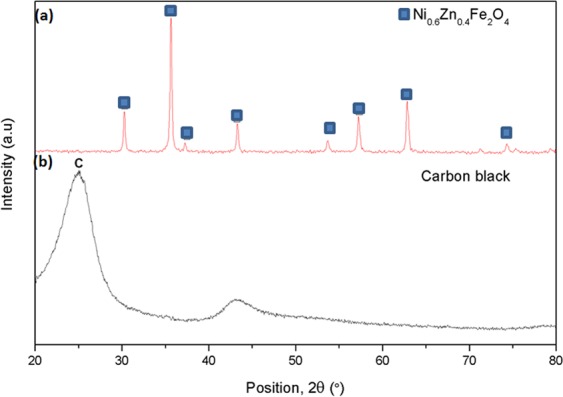
XRD spectra of (**a**) Ni_0.6_Zn_0.4_Fe_2_O_4_ and (**b**) carbon black.

The phase analysis was carried out by XRD measurements on sintered Ni_0.6_Zn_0.4_Fe_2_O_4_ and carbon black samples and the diffracted patterns are shown in Fig. 4. The carbon black exhibited poly-aromatic feature with characteristic carbon, C, observed at (002) peak. Some amorphous structures may coexist as suggested by the broad full half-peak width maximum (FWHM). The diffracted peaks of sintered Ni_0.6_Zn_0.4_Fe_2_O_4_ imply that the sample demonstrated fully crystalline spinel phase as shown by the sharp and high intensity peaks. The main peaks of Ni_0.6_Zn_0.4_Fe_2_O_4_ (JCPDS No.: 01-087-2338) are located at 2θ = 30.27°, 35.63°, 37.27°, 43.29°, 57.21° and 62.80°. Formation of Ni_0.6_Zn_0.4_Fe_2_O_4_ single phase is an important property in offering good absorption properties due to higher magnetic mass of the crystalline spinel structure over amorphous phase, leading to higher magnetization, thus resulting in better electromagnetic absorption.

### Electromagnetic properties

**Figure 5:**
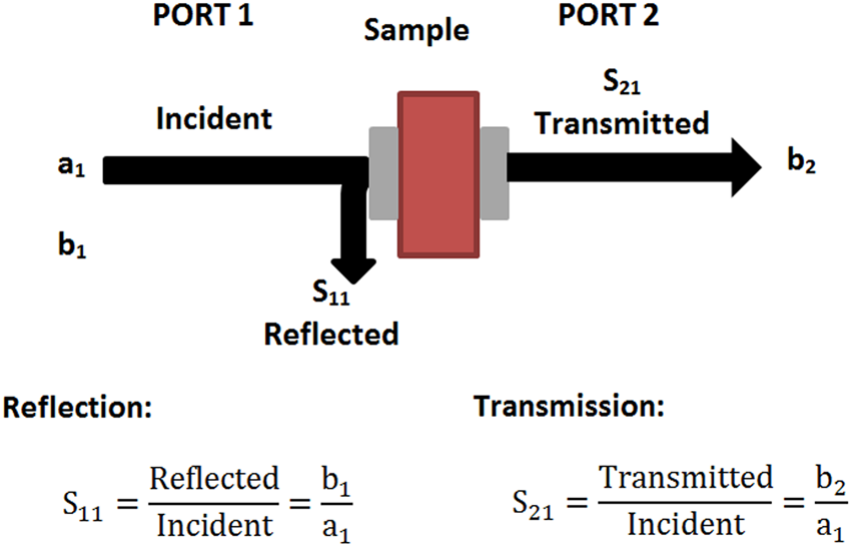
Schematic diagram of the reflection/transmission mechanisms which occur in a sample under test in the waveguide measurement setup.

Microwaves dissipate as heat within the material through various phenomena/mechanisms in the event of an incident on a loss dispersive material. A microwave absorber’s basic principle is to “consume” electromagnetic wave energy by increasing the mechanisms of loss in them. These materials are typically made up of dielectric or magnetic fillers and polymer in which the absorbing performance of the microwave is mainly affected by the fillers’ magnetic and dielectric properties, as well as the interactions between particles through the polymer matrix. Thus, the dissipation or loss mechanism is attributable to two material parameters: the complex relative dielectric permittivity (ε*) and the complex relative magnetic permeability (µ*). These two parameters are expressed through the following equations: µ* = µ′ − *j*µ″ and ε* = ε′ − *j*ε″ in which µ′ and ε′ are the real part of complex permeability and permittivity respectively and µ″ and ε″ are the imaginary parts. The real part indicates energy storage, while the imaginary part indicates energy loss or dissipation through the system, usually by conduction and resonance loss. The µ* and ε* values of the samples were calculated from the reflected (S_11_) and transmitted (S_21_) signals measured using a waveguide technique set up in our laboratory - the schematics of the reflection/transmission mechanisms is shown in Fig. 5. Details of the method have been published elsewhere^39^.

### Complex permeability

**Figure 6:**
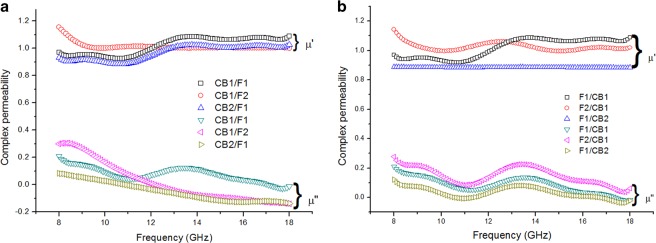
Complex permeability of (**a**) carbon black/epoxy resin as a matching layer and Ni_0.6_Zn_0.4_Fe_2_O_4_/epoxy resin as an absorbing layer; and (**b**) Ni_0.6_Zn_0.4_Fe_2_O_4_/epoxy resin as a matching layer and carbon black/epoxy resin as an absorbing layer.

From Fig. 6(a), the μ′ of carbon black as a matching layer and Ni_0.6_Zn_0.4_Fe_2_O_4_ as an absorbing layer ranges from ~0.9 to 1.2 while μ″ varies from less than zero to 0.3. The negative μ″ values exhibited by the present samples has been observed in many composite systems previously by other workers, such as reduced graphene oxide and graphene oxide^40^, multi-walled carbon nanotube composite^41^, Fe_3_O_4_/SnO_2_ core/shell nanorod^42^ and FeCo/C/Fe_2.5_Cr_0.5_Se_4_ nanocomposites^43^. This might be attributed to the phase lag between capacitance and inductance in the system as described in the equivalent circuit model proposed in^43^. It was observed that when carbon black acted as the matching layer and Ni_0.6_Zn_0.4_Fe_2_O_4_ as the absorbing layer, only the samples with total thickness of 2 mm showed a broad hump from 11 to 18 GHz, somewhat indicating the occurrence of resonance loss, while resonances of thicker samples might have occurred at lower frequencies, as indicated by the decreasing trends of µ″ in the graph.

A similar range of μ′ and μ″ values was observed in samples with ferrite as a matching layer and carbon black as the absorbing layer as shown in Fig. 6(b). A broad hump was however observed in μ″ plots for all of the samples. The μ″ plots correspond to magnetic losses where these losses are dominant in a ferrite. In such materials, the magnetic losses originate from various mechanisms such as magnetic hysteresis, eddy current losses, domain wall displacement and magnetic resonance^44^. As mentioned previously, the average particle size of Ni_0.6_Zn_0.4_Fe_2_O_4_ in this work is 53 nm which is much lower than the critical size for the domain wall formation, thus magnetic loss arising from domain wall displacement can be neglected. Therefore, the magnetic loss is mostly due to spin resonance, and since Ni_0.6_Zn_0.4_Fe_2_O_4_ is an insulator, the eddy current contribution is also not entirely significant but is frequency dependent. Generally, our samples show that μ″ decreased with frequency within the range of 8–15 GHz, meanwhile at the higher frequencies of 15–18 GHz the response showed almost a plateau-like trend. This will be explicitly explained later by the plots in Fig. 9 in which the dominant contribution of the losses over frequency can be inferred and demonstrated by the plots.

### Complex permittivity

There are generally four mechanisms of polarization that contribute to permittivity in dielectric materials – their contribution can be written as follows:

3 $${P}={{P}}_{{s}}+{{P}}_{{d}}+{{P}}_{{a}}+{{P}}_{{e}}$$

where *P* is the net polarizability, *P*_*s*_ is space charge polarizability, *P*_*d*_ is dipolar polarizability, *P*_*a*_ is atomic polarizability and *P*_*e*_ is electronic polarizability. In particular, electronic resonant frequency is within visible light, while resonant frequency for atomic polarization is within the infrared or far infrared region. Because microwave frequencies are well below the resonant frequencies of electronic and atomic polarizations, the permittivity resulting from such polarizations is almost independent of the frequency^45^. Therefore, their contributions to the ε′ and ε″ magnitudes are small in comparison to space charge and dipolar polarizations.

**Figure 7:**
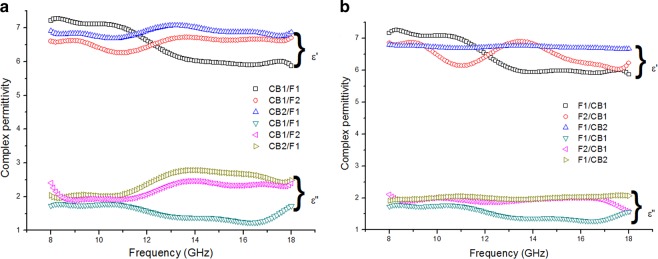
Complex permittivity of (**a**) carbon black/epoxy resin as a matching layer and ferrite/epoxy resin as an absorbing layer; and (**b**) ferrite/epoxy resin as a matching layer and carbon black/epoxy resin as an absorbing layer.

The plots of complex permittivity with frequency measured from our samples are shown in Fig. 7 in which maximum values of 7.3 and 3.0 for ε′ and ε″ were observed respectively. CB1/F1 sample showed almost stable ε′ values until 11 GHz, beyond which the values begin to drop. This similar trend was observed in F1/CB1 even when the ferrite acted as a matching layer and carbon black acted as an absorbing layer. The values of ε′ were almost independent of frequency for samples F1/CB2, but a very weak and broad hump was observed in F2/CB1, CB2/F1 and CB1/F2 samples.

The broad peaks of ε″ in the frequency range of 11–18 GHz as shown in Fig. 7(a) were observed in samples having total thickness of 3 mm, with carbon black acting as the matching layer and ferrite as the absorbing layer. However, a broad peak for CB1/F1 was not seen within this frequency range – this is expected to occur at higher frequencies since the plot begin to rise at 17 GHz. The broad peaks observed when ferrite acted as an absorbing layer were due to dielectric relaxation, caused by intrinsic dipolar polarization which come from positive anions surrounded by the negative O^2−^ in Ni_0.6_Zn_0.4_Fe_2_O_4_, as well as interfacial polarization. This is because ferrite and carbon black particles were separated by insulating matrix molecules, thus giving rise to heterogeneity. This would result in some space charge accumulating at the interface, which in turn would generate interfacial polarizations^15,16^. As the polarization charge cannot keep up with the variation of the external electromagnetic field, dielectric relaxation is present, resulting in higher microwave absorption in the composites. Nonetheless, the broadened peaks within this range of frequency are hardly seen when the carbon black acted as the absorbing layer, thus showing carbon black is working better as a matching layer. The small percentage of carbon black filler might be attributed to ineffective EM wave attenuation since the tunnel effect functions mostly among conductive particles where the spaces among the neighbouring particles are large^30^. It can be observed, too that ε″ reached almost 3 when carbon black was employed as the matching layer, while in contrast the value only reached 2 when carbon black acted as an absorbing layer. Hence, samples having carbon black as a matching layer and Ni_0.6_Zn_0.4_Fe_2_O_4_ as the absorbing layer with a total thickness of 3 mm show higher dielectric loss.

### Loss tangent

**Figure 8:**
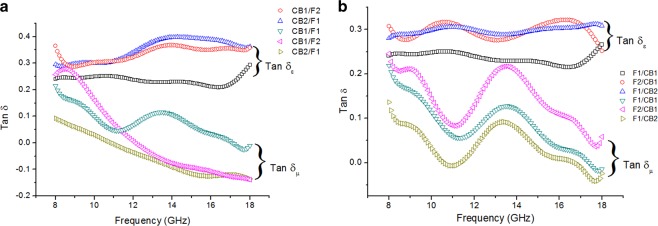
Loss tangent of (**a**) carbon black/epoxy resin as a matching layer and ferrite/epoxy resin as an absorbing layer; and (**b**) ferrite/epoxy resin as a matching layer and carbon black/epoxy resin as an absorbing layer.

From the complex permeability and permittivity plots in Figs. 6 and 7, the magnetic loss tangent, *Tan δ*_*µ*_, and dielectric loss tangent, *Tan δ*_*ε*,_ have been plotted (see Fig. 8) to observe the dominant loss contribution in the materials. The loss tangents are obtained by calculating the ratio of imaginary to real part of permeability and permittivity as follows:

4 $$Tan\,{\delta }_{\mu }=\frac{\mu {\prime\prime} }{\mu {\prime} };$$

5 $$\,Tan\,{\delta }_{\varepsilon }=\frac{\varepsilon {\prime\prime} }{\varepsilon {\prime} }$$

This quantity takes into account the overall microwave dielectric and magnetic losses inside the composite material under test. The higher its value, the greater are the losses.

**Figure 9:**
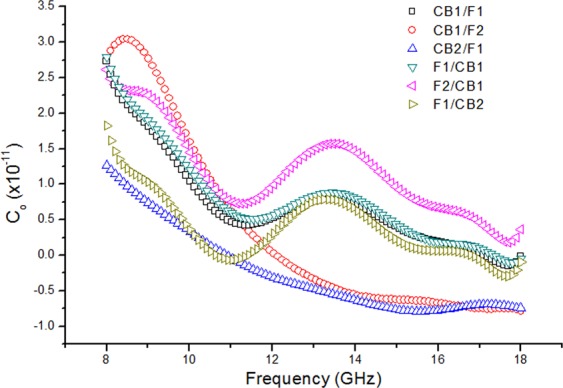
Eddy current effect of the double-layer composites from 8 to 18 GHz.

**Figure 10:**
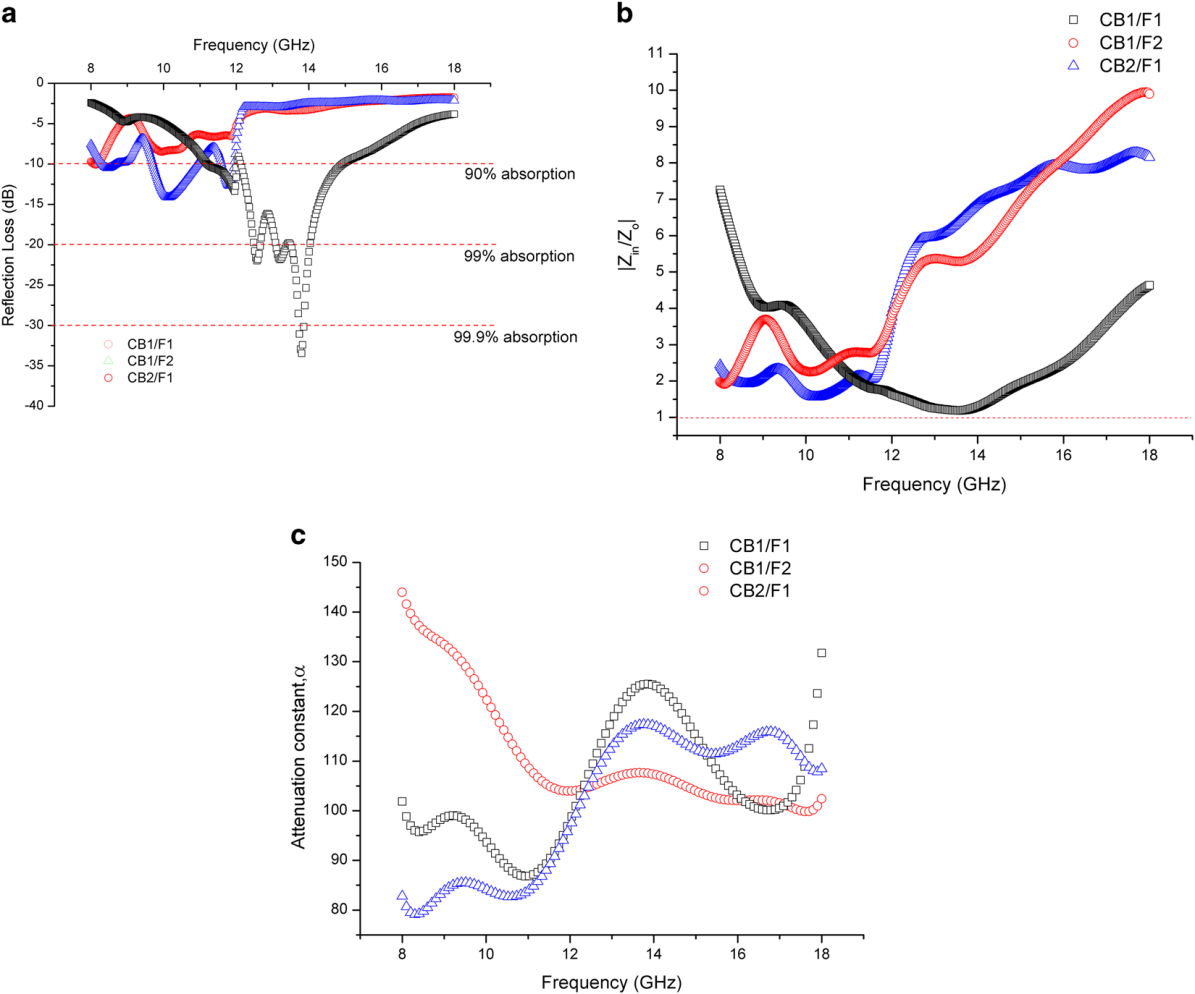
(**a**) Reflection Loss, (**b**) Normalized input impedance, |*Z*_*in*_/*Z*_*o*_| and (**c**) Attenuation constant versus frequency for carbon black/epoxy resin as a matching layer and ferrite/epoxy resin as an absorbing layer.

High loss tangent represents the capability of converting microwave to other forms of energy; therefore high loss tangent values are desired for a microwave absorber. Optimized complex permittivity is desired for a microwave absorber since low ε′ and ε″ would result in inefficient microwave attenuation, but if the values are too high impedance matching can be difficult^46^. It can be seen from Fig. 9 that generally the *Tan δ*_*ε*_ values are larger than *Tan δ*_*µ*_. The observed *Tan δ*_*ε*_ reached up to 0.40 at ~14 GHz and stayed higher than 0.35 until 18 GHz for sample CB2/F1 and 0.33 for F2/CB1. The values did not vary much for the samples having total thickness of 3 mm, however there is some difference in the values of *Tan δ*_*ε*_ among the 2 mm samples. Most of the plots show unbalance between the *Tan δ*_*ε*_ and *Tan δ*_*µ*_, however it could be observed that the distribution of the values for both plots of CB1/FI are relatively closer for all the samples, indicating better EM matching, thus exhibiting high performance of microwave absorption^47^. This could be due effective complementarities between permittivity and permeability, which is also seen in Fig. 10 since effective complementarities are essential in achieving efficient absorbing capability^42,48^. The much higher *Tan δ*_*ε*_ as compared to that of *Tan δ*_*µ*_ demonstrates that the dielectric loss dominates the attenuation of EM energy over the frequency range.

### Microwave conductivity

At microwave frequencies, eddy current and ferromagnetic resonance are factors that contribute to magnetic loss. Eddy current effect is described through the following relation^49^:

6 $${C}_{o}=\frac{\mu {\prime\prime} }{{(\mu {\prime} )}^{-2f-1}}=[2\pi {\mu }_{o}\sigma {d}^{2}]/3$$

where *µ*_*o*_ is the permeability of vacuum and *µ*′ and *f* are as defined previously, σ is the conductivity and *d* is the sample thickness. If the observed magnetic loss is only due to eddy current, the quantity of *C*_*o*_ should be constant when the frequency varies^50^.

Fig. 9 shows plots of *C*_*o*_ with frequency for the composite structures. The values of the quantity for all the samples fluctuated quite distinctly with frequency, except CB1/F2 and CB2/F1. These results indicated there is just a small contribution from the eddy current loss and it does not play a major role in the magnetic loss within this range of frequency. The *C*_*o*_ for the CB1/F2 and CB2/F1 samples decreased with frequency and showed a more stable behaviour and plateau-like trend beyond about 14 GHz, ascribing the phenomenon of eddy current effect. Using the above theory we can therefore deduce that the resonance in all of the samples were principally due to natural ferromagnetic resonance within the frequency range. Meanwhile starting from 14 GHz and above, eddy current loss started to dominate in CB 1 mm/F 2 mm and CB 2 mm/F 1 mm samples.

### Microwave absorption properties

The microwave absorption ability of a material is dependent on two important factors. First, the material needs to possess good impedance matching characteristics and secondly, it needs to have a strong attenuation capability. The latter is closely related to complex permittivity, complex permeability, specific conductance, thickness, and absorber structure^51^. This is to ensure that EM waves could penetrate the materials efficiently with minimal reflection at the interfaces. The reflections are reduced if the characteristic impedance of the absorber is closely matched to that of free space.

For a single-layer microwave absorber, the reflection loss can be expressed as a function of the normalized input impedance of a metal-backed absorber. The normalized input impedance (*Z*_*in*_) of such a system is given by:

7 $${Z}_{in}=\frac{{Z}_{i}}{{Z}_{o}}=\sqrt{\frac{{\mu }_{r}}{{\varepsilon }_{r}}}tanh(\frac{j2\pi \sqrt{{\mu }_{r}{\varepsilon }_{r}}}{c}\,fd)$$

here *Z*_*in*_ is the normalized input impedance at the absorber surface, *Z*_*i*_ is the input impedance, *Z*_*o*_ is the free space impedance and *d* is the thickness of absorber, while ${{\rm{\mu }}}_{{\rm{r}}}$ and ${{\rm{\varepsilon }}}_{{\rm{r}}}$ are the relative permeability and permittivity of the medium, and *f* and *c* are as defined previously. The reflection loss is a ratio of reflected power to that of incident power, it is related to Z_in_ as follows [13]:

8 $${Reflection}\,{loss}\,({dB})=20\,\log \,{}_{10}[\frac{({Z}_{in}-1)}{({Z}_{in}+1)}]$$

Double-layer absorbers can be classified as graded inter-facial impedance matching components. An EM wave impinging upon an interface will experience some reflection that is proportional to the magnitude of the impedance mismatch between incident and transmitting media. The impedance transition between the layers in such a material is typically gradual so that the reflection is reduced. This requires that the front interface with air must be of a low loss material and the material for the following layer must be lossier than the previous layer^3^. For a double-layer microwave absorber, the input impedance *Z*_*in*_ can be calculated according to the following equation^51^:

9 $${Z}_{in}=\frac{\sqrt{\frac{{\mu }_{2}}{{\varepsilon }_{2}}(\sqrt{\frac{{\mu }_{1}}{{\varepsilon }_{1}}}tanh[j(\frac{2\pi f{d}_{1}}{c})\sqrt{{\mu }_{1}{\varepsilon }_{1}}]+\sqrt{\frac{{\mu }_{2}}{{\varepsilon }_{2}}tanh[j(\frac{2\pi f{d}_{2}}{c})\sqrt{{\mu }_{2}{\varepsilon }_{2}}]})}}{\sqrt{\frac{{\mu }_{2}}{{\varepsilon }_{2}}+\sqrt{\frac{{\mu }_{1}}{{\varepsilon }_{1}}}tanh[j(\frac{2\pi f{d}_{1}}{c})\sqrt{{\mu }_{1}{\varepsilon }_{1}}]}tanh[j(\frac{2\pi f{d}_{2}}{c})\sqrt{{\mu }_{2}{\varepsilon }_{2}}]}$$

where ${d}_{1}$, ${d}_{2}$, ${\varepsilon }_{1}$, ${\varepsilon }_{2}$, ${\mu }_{1}$, and ${\mu }_{2}$ are the thicknesses, relative complex permittivity, and complex permeability of Layers 1 and 2, respectively. The schematics of a metal-backed double-layer absorber are illustrated in Fig. 1. Layer 1 refers to the absorbing layer, while Layer 2 refers to the matching layer. When the incident EM wave irradiates the absorber surface, it should propagate to the matching layer with minimal reflection, and ideally be consumed in the absorption layer. Based on this presumption, a double-layer absorber should be able to offer more absorption, and over a broader bandwidth, owing to interfacial polarization at the interfaces of the heterogeneous media in the structure, thus contributing to enhanced microwave absorption performance.

The attenuation constant *α* is used to quantify attenuation – it can be expressed in terms of the dielectric and magnetic losses in an absorber using the following equation:

10 $$\alpha =\frac{\sqrt{2}\pi f}{c}\times \sqrt{(\mu {\prime\prime} {\rm{\varepsilon }}{\prime\prime} -\mu {\prime} \varepsilon {\prime} )+\sqrt{{(\mu {\prime\prime} {\rm{\varepsilon }}{\prime\prime} -\mu {\prime} \varepsilon {\prime} )}^{2}+{(\mu {\prime} \varepsilon {\prime\prime} -\mu {\prime\prime} \varepsilon {\prime} )}^{2}}}$$

**Figure 11:**
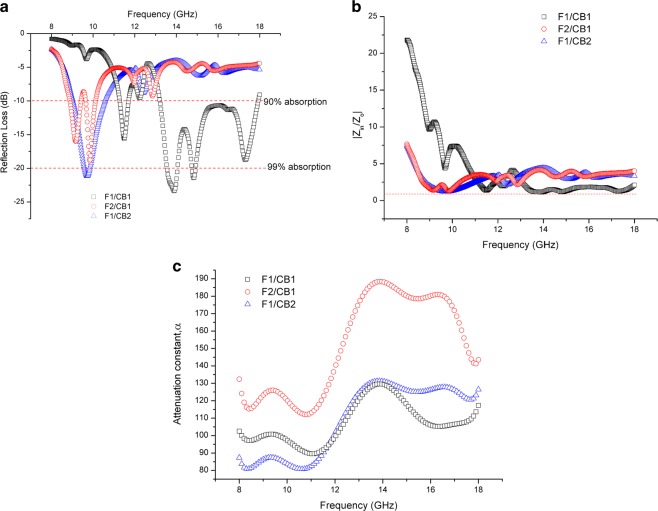
(**a**) Reflection Loss, (**b**) Normalized input impedance, |Z_in_/Z_o_| and (**c**) Attenuation constant versus frequency for ferrite/epoxy resin as a matching layer and carbon black/epoxy resin as an absorbing layer.

The reflection loss, normalized input impedance and attenuation constant for all the samples measured are shown in Figs. 10 and 11. It can be seen that beyond 11 GHz, both of CB1/F1 and F1/CB1 demonstrated a significant absorption (Figs. 10(a) and 11(a)). Sample F1/CB1 in which carbon black acted as the absorbing layer offered a significant microwave absorption performance of about 4.8 GHz at −10 dB absorption bandwidth (*i.e*. 90% absorption), compared to CB1/F1. Even though CB1/F1 showed a higher attenuation of nearly −35 dB, the −10 dB bandwidth was only 2.7 GHz. The absorption properties in CB1/F1 were still significant in which more than 99% energy was absorbed. The broader bandwidth of 4.8 GHz observed in F1/CB1 as compared to that of CB1/F1 was due to better impedance matching between the front absorber and free space, thus minimizing reflection at the front-face. The impedance matching ensures that the EM waves enter the absorber optimally. That is, the absorber input impedance (*Z*_*in*_) and free space (*Z*_*o*_) should satisfy |*Z*_*in*_*/Z*_*o*_| = 1. Here, the |*Z*_*in*_*/Z*_*o*_| values were nearly 1 from 13 to 14 GHz for CB1/F1 (Fig. 10(b)), while when the layers were reversed; the matching is broader from 13 to 17.5 GHz (Fig. 10(b)).

The attenuation constant values α for all the samples were computed using Eq. (10) – they are plotted in Figs. 10(c) and 11(c). The following points can be deduced by tuning the matching-absorbing layer arrangement:

1. When the layers were arranged with CB matching layer/ferrite absorbing layer:
  (i) Sample CB1/F1 showed the strongest absorption of more than 99.9% absorption, with α reaching almost 125 around 13.8 GHz. The same sample also demonstrated the best impedance matching at around the same frequency – this proves a strong correlation between absorption and impedance matching, as expected from theory. Also, as predicted by theory too, the attenuation constant α for this sample was the highest at that frequency among all the samples tested.
  (ii) Below 12 GHz, sample CB1/F2 showed the most improvement among all the samples in terms of its attenuation, with α reaching up to about 145 at 8 GHz. However, due to poorer impedance match of this sample compared to sample CB2/F1, the absorption properties are strongest in CB2/F1 below 12 GHz.
2. When the layer arrangements were reversed:
  (i) All samples showed improvement in reflection loss, with all samples now showing more than 90% absorption. This is in contrast to the previous condition before the layers were reversed, where only the CB1/F1 and CB2/F1 samples achieved more than 90% absorption
  (ii) The bandwidth increased for all the samples (Fig. 11(a)), with impedance matching improved significantly, showing flatter responses from 9 to 18 GHz (Fig. 11(b)). The attenuations were also higher for all samples, with sample F2/CB1 showing the best α of about 190 (Fig. 11(c)).

For thicker samples with total thickness of 3 mm, the following observations were found:

1. Strongest absorptions were shown by F2/CB1 and F1/CB2 samples from 9 to 10 GHz, displaying nearly 99% and more than 99% absorption rates respectively (Fig. 11(a)), with corresponding good impedance matching in the said frequency band (Fig. 11(b)).
2. When the layers were swapped, the absorption largely decreased from more than 99% to about 90% for CB2/F1, while sample CB1/F2 barely offered 90%.
3. The reason for this reduction was mostly attributed to impedance mismatch (Fig. 10(b)), resulting in the EM waves to be reflected from the front surface of the composite structure even though significant loss tangent (Fig. 8) and large attenuation constant values (Fig. 10(c)) were observed in the samples. However when the layers were swapped, better impedance matching was observed within the same frequency range.

The frequency at which the reflection loss is minimum (*f*_*m*_) also shifted with increasing thickness: for the same ferrite thickness, increasing the carbon black layer from 1 mm (F1/CB1) to 2 mm (F1/CB2) shifted *f*_*m*_ to a lower frequency, while the – 10 dB bandwidth decreased from 4.8 to 1.6 GHz. The resonant frequency shifts correspond to the matching layer thickness *t*_*m*_ as predicted by the following equation

11 $${t}_{m}=\frac{nc}{4{f}_{m}\sqrt{|{\mu }_{r}{\varepsilon }_{r}|}},\,n=1,3,5\ldots $$

According to a literature^27^, increasing the carbon black content will enhance the dielectric polarization while more surface areas are available to attenuate electromagnetic wave by multi-scatter and reflection. Furthermore, more conductive particles per unit volume will enhance the conductive network, therefore attenuating the waves by eddy current. However, in this study, the thinner sample with 1 mm thickness of carbon black (F1/CB1) showed a better performance of microwave absorption properties which might be due to better impedance matching from the double-layer structure showed in Fig. 11(b), as the ferrite layer acted as the matching layer. It is interesting to note that, when the thickness of the matching and absorbing layer changed, but the overall thickness of the double-layer composites stayed the same, the microwave absorption performance is also different, thus signifying the roles of the matching and absorbing layers. The absorption parameters of all the samples are summarized in Table 2. To show more detail comparison of the absorbing properties; other related microwave absorbers are also compared. Compared with other carbon-based and ferrite materials with single, double and multi-layer structures as presented in Table 2, this work demonstrated relatively high microwave absorption performance in terms of the substantial microwave absorption with broad absorption bandwidth. Notably, the sample in the present work displays a remarkable broad absorption bandwidth of 4.8 GHz, which is wider than that of other materials.

**Table 2:** Microwave absorption properties of the double-layer nanocomposites and comparison with other related microwave absorbers.

Sample	Types of structure	Total thickness (mm)	Minimum reflection loss (dB)	RL_min_ Frequency (GHz)	−10 dB Bandwidth (GHz)	Reference
CB1/F1	Double-layer	2	−33.8	13.8	2.7	In this work
CB1/F2		3	−10.5	8.1	0.3	
CB2/F1		3	−13.8	10.1	1.3	
F1/CB1		2	−24.0	13.9	4.8	
F2/CB1		3	−18.8	9.9	0.6	
					0.6	
F1/CB2		3	−23.8	9.8	1.6	
NiFe_2_O_4_/Ni_0.6_Zn_0.4_Fe_2_O_4_		1.72	−39.0	9.4	2.8	^52^
5.5 wt.% CB doped SiO_2f_/PI (matching) /15 wt.%CB doped SiO_2f_/PI (absorbing)					~4.0	^53^
		1.6	−46.2	16.1		
MWCNTs/PVC	Multi-layer (8 layers; CNT-0 and CNT-10 layers were arranged alternately)				3.0	^54^
		2	−30.6	10.5		
5% CB	Single-layer	1	−23.2	8.5, 10.1	~0.3	^27^
					~0.1	
Ni_0.6_Zn_0.4_Fe_2_O_4_		2	−12.0	3.4	2.5	^55^
Ni_0.6_Zn_0.25_Co_0.15_Fe_2_O_4_		2	−15.2	5.4	3.6	
Ni_0.6_Zn_0.25_Cu_0.15_Fe_2_O_4_		2	−13.2	3.5	2.6	
Ni_0.6_Zn_0.4_Fe_2_O_4_		2	−11.2	11.0	2.4	^56^
MWCNT/Ni_0.5_Zn_0.5_Fe_2_O_4_		3	−19.3	8.5	1.2	^57^
MnNiZn ferrite PANI(1:1)/Paraffin		3	−31.3	11.1	3.7	^58^

## Conclusions

Double-layer composite absorbers have been fabricated via high energy ball milling with subsequent sintering and drop casting technique. The effects of the layer positions of the absorbers on their electromagnetic and microwave absorption properties were systematically studied by tuning the matching and absorbing layers. The CB1/F1 sample demonstrated the largest microwave absorption of more than 99.9% with minimum reflection loss of −33.8 dB, but with an absorption bandwidth of only 2.7 GHz. Significant enhancements of microwave absorption properties were observed in F1/CB1 sample. Due to better impedance matching and higher attenuation capability, the F1/CB1 sample showed the best all round performance, in which more than 99% microwave energy was absorbed, with a reflection loss of −24.0 dB and a widest bandwidth of 4.8 GHz at −10 dB, yet it is the thinnest among the three designs, having a total thickness of only 2 mm.
